# Clinical features and incidence of visual improvement following systemic antibiotic treatment in patients with syphilitic uveitis

**DOI:** 10.1038/s41598-022-16780-5

**Published:** 2022-07-22

**Authors:** Wantanee Sittivarakul, Sukrita Aramrungroj, Usanee Seepongphun

**Affiliations:** Department of Ophthalmology, Faculty of Medicine, Prince of Songkla University, 15 Karnjanavanich Rd, Hat Yai, Songkhla, 90110 Thailand

**Keywords:** Diseases, Health care, Medical research, Neurology, Signs and symptoms

## Abstract

To describe the clinical features, longitudinal pattern, and incidence rate of improvement of visual acuity (VA) following antibiotic therapy in patients with syphilitic uveitis. A total of 36 patients were included in this retrospective study from 2009 to 2020. The longitudinal patterns of mean VA values during follow-up were analyzed using a linear mixed model. Most patients were men with HIV coinfection (81%) and presented with panuveitis (49%). The mean VA at baseline improved from 0.97 to 0.39 logMAR at 6 months and remained stable thereafter. The cumulative incidence of VA ≥ 20/25 achieved by 2 years was 70%. Receiving antibiotic therapy within four weeks of the onset of ocular symptoms (adjusted hazard ratio [aHR] 3.4, *P* = 0.012), absence of HIV coinfection (aHR 8.2, *P* < 0.001), absence of neurosyphilis (aHR 6.5, *P* = 0.037), better presenting VA (aHR 5.0, *P* = 0.003), and intermediate uveitis as opposed to panuveitis (aHR 11.5, *P* = 0.013) were predictive of achieving VA ≥ 20/25. Men with HIV coinfection represented the majority of our patients. Visual outcomes, in response to antibiotics, were favorable. Delayed treatment, poor presenting VA, presence of HIV coinfection, and concomitant neurosyphilis decreased the likelihood of VA restoration.

## Introduction

Syphilis is a common sexually transmitted infection (STI) caused by the bacterium *Treponema pallidum* (*T. pallidum*). The organism has the ability to infect multiple organ systems, including the eyes. All parts of the ocular structure can be involved; however, uveitis accounts for the majority of ocular presentations and can manifest in both early- and late-stage syphilis^1,2^. A global increase in the incidence of syphilis has been observed for the past few decades. In 2016, approximately 6.3 million new cases were recorded worldwide^3^. In the United States and Western Europe, the high-risk population are men who have sex with men (MSM), especially those with coexisting human immunodeficiency virus (HIV) infection^4,5^. Consequently, an increase in the incidence of ocular syphilis has been observed with the re-emergence of systemic infection^1,6–9^.

The risk of ocular syphilis is relatively low; it comprises approximately 0.6–2.7% of total syphilis infections^1,6^. However, visual acuity (VA) may be severely affected once ocular tissue is involved^8,10–12^. Prompt antibiotic treatment is associated with a more favorable visual prognosis than delayed treatment. Approximately 60–80% of eyes were able to gain two or more Snellen lines of VA with prompt treatment^8,13,14^. Poor visual prognosis is associated with delayed treatment, poor VA at presentation, inadvertent systemic corticosteroid use prior to syphilis treatment, and HIV coinfection^12,13,15–18^.

Likewise, a resurgence of syphilis has been reported in Thailand. A five-fold increase in the annual prevalence of syphilis was observed from 2008 to 2018, increasing from 2.16 to 11.51 cases per 100,000 population^19^. However, information on ocular syphilis in Thai patients is lacking. In addition, limited studies have been carried out to estimate the incidence of visual improvement following systemic antibiotic treatment. The present study aimed to describe the demographics, clinical presentation, and laboratory findings of patients with syphilitic uveitis, with and without HIV coinfection. Additionally, the longitudinal pattern of VA and predictive factors associated with achieving maximum VA of ≥ 20/25, a level considered to be within the range of normal vision^20^, were investigated.

## Methods

### Study population

This retrospective study was approved by the Ethics Committee of the Faculty of Medicine, Prince of Songkla University (REC number 62-046-2-4). The Ethics Committee of the Faculty of Medicine, Prince of Songkla University determined that written informed consent was not required for this study, as the risks involved were minimal and a waiver would not adversely affect the rights and welfare of the study participants. Patient data was kept confidential and the Declaration of Helsinki was followed.

A retrospective chart review was performed in all patients with syphilitic uveitis managed at Songklanagarind Hospital, a major tertiary center in southern Thailand, between January 2009 and December 2020. The diagnosis of syphilitic uveitis was based on the classification criteria developed by the Standardization of Uveitis Nomenclature (SUN) working group; these criteria consist of a compatible clinical history, uveitic presentation, and serologic confirmation of systemic infection with *T. pallidum*^21^. In addition, a clinical response must be observed following systemic antibiotic treatment for syphilis. Positive syphilis serologic testing was defined as reactive initial treponemal tests, including chemiluminescent immunoassay or fluorescent treponemal antibody absorption (FTA-Abs), in addition to either reactive nontreponemal testing including rapid plasma reagin or venereal disease research laboratory (VDRL) or reactivity on another different treponemal test. Patients with the following characteristics were excluded from the study: age < 18 years, having a history of diagnosed syphilis and adequate syphilis antibiotic treatment, those who were lost to follow-up before receiving antibiotic treatment for syphilis, and those who had uveitis with positive syphilis serology but demonstrated no response to syphilis antibiotic treatment. Patients were initially identified from the uveitis electronic database system created for clinical record keeping for all new consecutive patients seen at our uveitis clinic. The medical records of the identified patients were subsequently reviewed to include only the individuals who fulfilled the inclusion criteria.

### Data collection

The data collected included age, sex, laterality, duration between onset of ocular symptoms and syphilis treatment, and systemic symptoms of syphilis. Ophthalmologic examination results included best corrected visual acuity (BCVA) using the Early Treatment of Diabetic Retinopathy Study chart with logMAR conversion, and intraocular pressure (IOP) measured by Goldmann applanation tonometry. The anatomic location of uveitis and cellular grading were categorized according to the SUN guidelines and classified as anterior, intermediate, posterior, or panuveitis^22^. The following ocular complications were recorded at presentation and during follow up; ocular hypertension (IOP > 24 mmHg), hypotony (IOP < 4 mmHg), cataract (≥ grade 1), retinal detachment (RD), optic atrophy, epiretinal membrane (ERM), and cystoid macular edema (CME), which were confirmed by optical coherence tomography (OCT).

Laboratory data included syphilis serologic test results, anti-HIV antibody results, CD4 counts, and cerebrospinal fluid (CSF) examination results obtained through a lumbar puncture (LP). CSF abnormality was consistent with neurosyphilis when there was a reactive CSF-VDRL test, and/or a white blood cell (WBC) count > 5 cells/µL (or > 20 cells/µL in HIV-infected patients), or a protein reading > 50 mg/dL found in the CSF. These higher cut-offs used for the WBC count and protein were chosen because these values were above the mild pleocytosis and protein elevation due to the HIV infection itself; therefore, diagnostic specificity of neurosyphilis for HIV-infected patients was improved^23,24^. Additionally, treatment data included antibiotic regimens, adjunctive systemic corticosteroids, and antiretroviral therapy in patients who were co-infected with HIV. Intravenous (IV) penicillin G was the standard antibiotic treatment for all patients unless they were allergic to penicillin. For such patients, alternative regimens would be prescribed according to the infectious disease or neurology specialist’s suggestion. A lumbar puncture for CSF examination was routinely advised in all cases, regardless of the severity of intraocular inflammation or HIV infection status.

### Main outcome measures

The main outcome measure was the mean BCVA and the proportion of eyes with BCVA thresholds of ≥ 20/25 (range of normal vision), < 20/40 to > 20/200 (moderate visual loss, [MVL]), and 20/200 or worse (severe visual loss, [SVL]) at 1, 3 (± 2 weeks), and 6 (± 4 weeks) months; 1 year (± 6 weeks); and 2 years (± 8 weeks). Secondary outcome measures included the incidence rate of VA improvement to a level of ≥ 20/25 post-treatment. The analysis of VA outcomes was performed by eye. Eye-time at risk was calculated beginning from the initial presentation among eyes of patients presenting with a baseline BCVA < 20/25 and ending with either the date wherein the VA first achieved ≥ 20/25, or the last follow-up visit for eyes that did not achieve a VA of ≥ 20/25.

### Statistical analyses

**Figure 1:**
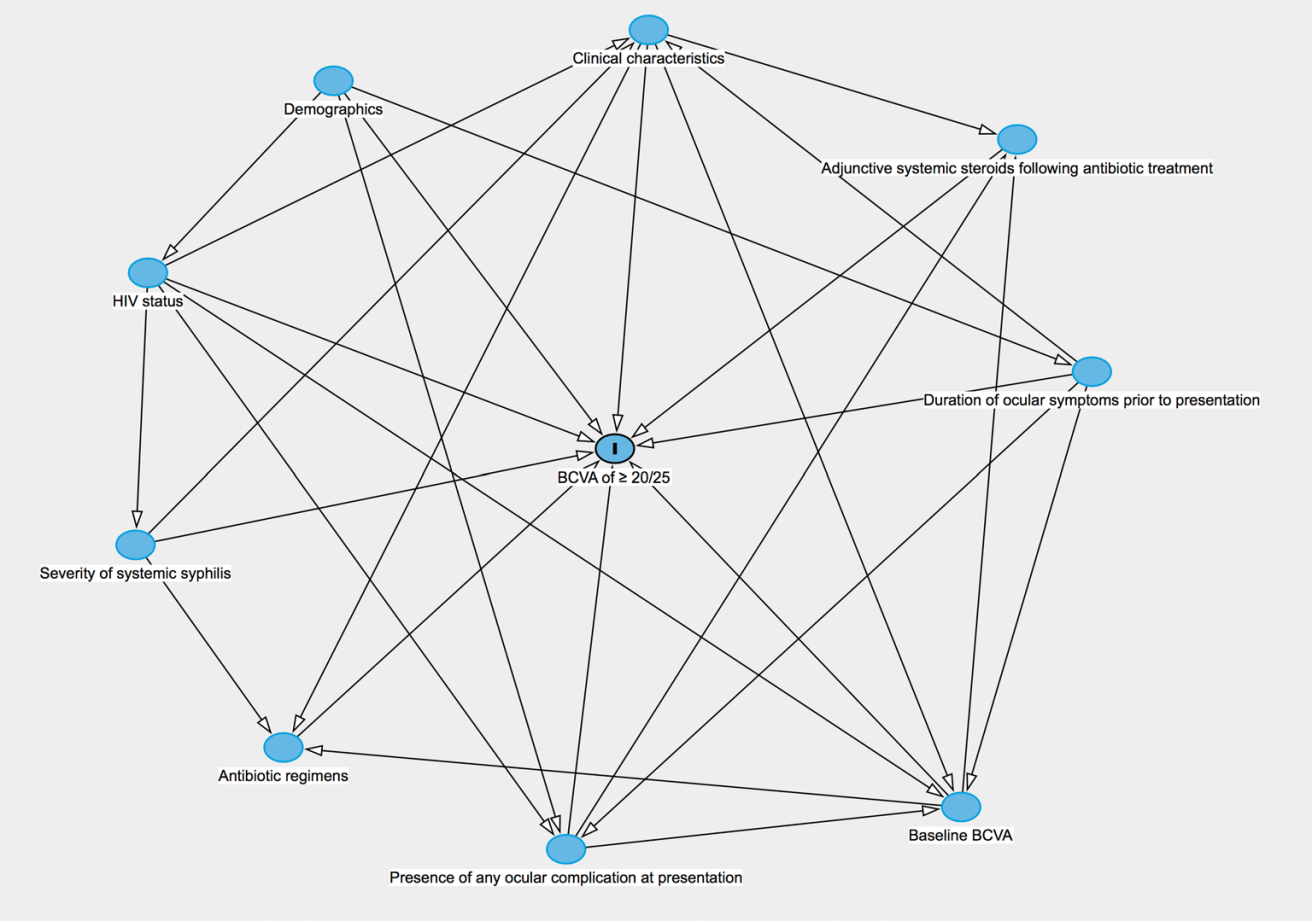
Theoretical model of the association between all variables of interest and improvement of visual acuity following antibiotic therapy based on a directed acyclic graph (DAG). BCVA, best corrected visual acuity; HIV, human immunodeficiency virus.

The statistical analyses were performed using STATA version 14 (StataCorp LP, College Station, TX, USA). Descriptive statistics were reported as frequencies (%), means (standard deviation), or medians (interquartile range, [IQR]). Pearson’s Chi-Square or Fisher’s exact test were used to analyze the relationship between categorical variables. The Mann–Whitney U test and Kruskal–Wallis test were used to analyze the relationship between continuous and categorical variables. The longitudinal patterns of mean BCVA values during follow-up were analyzed using a linear mixed model, in which the patient and eyes were considered as the random elements and time was the fixed effect. The incidence rate of VA improvement to ≥ 20/25 was calculated from the total number of events that developed among all affected eyes with baseline BCVA < 20/25 at initial presentation divided by the number of eye-months (EMs) at risk. Kaplan–Meier curves on the time to BCVA improvement to ≥ 20/25 and the log-rank test were performed. To determine factors associated with achieving a VA of ≥ 20/25, a directed acyclic graph (DAG) using DAGitty Version 3.0 (Johannes Textor, Utrecht University, NL) that included all domains of interest was constructed to identify bias-minimized models. The potential causal pathways between the variable domains and covariates were generated based on the understanding of the subject matter (Fig. 1). Based on the assumptions described in the DAG, a minimal set of covariate domains was selected to investigate the total effect of each exposure on achieving BCVA ≥ 20/25. Multivariate Cox regression models based on the DAG were performed using robust estimation of standard error to account for more than one eye per patient in the data set. Selection of variables within each domain was made using backward elimination of variables not contributing significantly to the fit of the model, guided by the change in the log likelihood of successive hierarchical models, retaining only those variables with a *P* value of < 0.05. In the modeling process, missing values were accounted for using the covariate adjustment method.

## Results

### Demographic and baseline characteristics of the study population

Initially, 41 patients diagnosed with uveitis and tested positive for syphilis on serologic testing during the study period were identified from the database. After a careful review of the medical records, five patients were excluded. Among them, two were lost to follow-up before receiving antibiotic treatment for syphilis, and three only had reactive FTA-Abs without reactive nontreponemal testing and with a history of syphilis which had been adequately treated with antibiotics. No patients were excluded due to a lack of response to antibiotic treatment for syphilis. Finally, 36 patients (65 eyes) were included in the study. The median follow-up time of the entire cohort after presentation at our clinic was 13.5 months (IQR 4–24.7).

Table 1 summarizes demographic and patient characteristics at presentation. All patients were Thai, and 35 of 36 patients (97%) were men. The median age at presentation was 36 years (IQR 29–41 years). No patient had a history of previously diagnosed or treated syphilis. Of the 26 men for whom data was recorded on their history of sex with other men, 21 (80%) confirmed this history. Twenty-nine patients (81%) had HIV coinfection; all were men. Of these 29 patients, 20 (69%) were newly diagnosed with HIV after the diagnosis of syphilitic uveitis. The median CD4 count at presentation in patients with HIV coinfection was 211 cells/µL (IQR 127-401 cells/µL). Thirteen patients (36%) had a history of systemic features of syphilis in which a rash involving the palms and soles was the most frequent manifestation (22%). Twenty-nine patients (80%) had bilateral uveitis.

**Table 1:** Demographic and clinical characteristics of 36 patients with syphilitic uveitis at presentation.

Characteristics	n (%), or Median (IQR)
Median age (years)	36 (29.25, 41)
**Sex**	
Male	35 (97.2)
MSM	21 (80.8)
Non-MSM	5 (19.2)
Unknown	9 (25.7)
Female	1(2.8)
**Laterality of uveitis**	
Unilateral	7 (19.4)
Bilateral	29 (80.6)
**Systemic features of syphilis either at or prior to enrollment**	
Genital chancre	7 (19.4)
Maculopapular rash on palms and soles	8 (22.2)
Alopecia	6 (16.7)
Oral ulcer	5 (13.9)
Arthritis	1 (2.8)
**Duration of ocular symptoms prior to presentation**	
Median	4.29 (2.36, 9.11)
< 4 weeks	10 (27.8)
4–12 weeks	18 (50.0)
> 12 weeks	8 (22.2)
Median serum nontreponemal titer	64 (32,128)
**HIV coinfection**	29 (80.6)
Already known	9 (31.0)
Newly- diagnosed	20 (69.0)
**Median CD4 counts (cells/μL)**^**a**^	211.5 (127, 401.5)
≤ 100	5 (17.2)
101–200	9 (31.0)
> 200	14 (48.3)
Missing	1 (3.5)
**Receiving ART at presentation**^**a**^	
Yes	7 (24.1)
No	22 (75.9)
**Lumbar puncture at enrollment**	
Performed	24 (66.7)
At least 1 abnormal value	20 (83.3)
CSF-VDRL reactive (total n = 16)	10 (62.5)
Median CSF-VDRL titer	1 (NR, 3.5)
Elevated CSF WBC^b^	14 (73.7)
Median CSF WBC/μL	33 (9, 82)
Elevated CSF protein > 50/dL	19 (79.2)
Median CSF protein (mg/dL)	72 (56, 92)
**Systemic antibiotics**	
Intravenous penicillin G	33 (91.7)
Ceftriaxone followed by doxycycline	2 (5.6)
Azithromycin followed by doxycycline	1 (2.8)

^a^Calculated in HIV-infected patients only.

^b^Considered abnormal if > 5/μL in HIV-uninfected patient, > 20/μL for HIV-infected patient.

ART: antiretroviral therapy; CSF: cerebrospinal fluid; MSM: men who have sex with men.

All patients had a reactive serum nontreponemal test with a median titer of 1:64 (IQR 32–128). Twenty-four patients (67%) underwent LP upon consultation. None of our patients reported neurological symptoms, other than ocular symptoms. A total of 20 (83%) out of 24 patients had CSF abnormalities that met our CSF criteria for neurosyphilis. Among them, 18 patients were HIV-infected and two were not (*p* = 0.018, Fisher’s exact test). CSF-VDRL results were available in 16 patients, of whom, ten (63%) were reactive. Elevated CSF WBC and protein were observed in 14 (74%), and 19 patients (79%), respectively. Elevated CSF protein concentration was the only CSF abnormality in four patients. Regarding antibiotic treatment, 33 patients (92%) received intravenous penicillin G (12–16 million units/day) for two weeks as the primary treatment regardless of CSF examination results or HIV status. The remaining three patients received other regimens due to penicillin allergy (IV ceftriaxone, two patients; and azithromycin, one patient). Following the completion of systemic antibiotic therapy, eight patients received oral prednisolone (20–30 mg/day with tapering and discontinuation within three months) owing to the presence of significant CME and/or vitritis (vitreous haze grade ≥ 2 +).

Ophthalmologic presentations of all affected eyes are presented in Table 2. Overall, the median presenting logMAR VA was 0.70 (Snellen equivalent = 20/100). Panuveitis was the most common anatomic location (49%). Optic disc edema/hyperemia (49%) and retinal vasculitis (39%) were the top two most common posterior segment findings. The most common ocular complications at presentation were serous RD and CME, which were equally observed in four (7.7%) of 65 eyes. During follow-up, new-onset CME was observed in three eyes. Cataract was noted in two eyes, in which pars plana vitrectomy had been previously performed to repair RD. Ocular hypertension, hypotony, ERM and optic atrophy were observed in one eye each.

**Table 2:** Clinical characteristics of 65 eyes with syphilitic uveitis at presentation.

Characteristics	Total	HIV-infected	HIV-uninfected	*P* value
	(N = 65)	(n = 52)	(n = 13)	
**Age (years)**				
Median (IQR)	36 (29, 41)	34 (29, 38.5)	41 (29, 53)	0.126
**Sex, n (%)**				
Male	63 (96.9)	52 (100)	11 (84.6)	0.038
Female	2 (3.1)	0 (0)	2 (15.4)	
Mean baseline logMAR BCVA (SD)	0.97 (0.85)	1.10 (0.86)	0.46(0.55)	0.003
Median baseline logMAR BCVA (IQR)	0.70 (0.3, 1.6)	0.89 (0.34, 1.90)	0.24 (0.16, 0.4)	0.005
**Baseline BCVA, n (%)**				
≥ 20/25	8 (12.3)	5 (9.6)	3 (23.1)	0.147
20/32–20/40	15 (23.1)	10 (19.2)	5 (38.4)	
20/50–20/160	18 (27.7)	15 (28.9)	3 (23.1)	
≤ 20/200	24 (36.9)	22 (42.3)	2 (15.4)	
**IOP, mmHg**				
Mean (SD)	12.3(4.27)	11.8(3.17)	14.0 (7.03)	0.091
Bilateral involvement, n (%)	58 (89.2)	46 (88.5)	12 (92.3)	0.689
**Anatomic location of uveitis, n (%)**				
Anterior	2 (3.1)	2 (3.8)	0 (0)	0.315
Intermediate Uveitis	15 (23.1)	10 (19.2)	5 (38.5)	
Posterior Uveitis	16 (24.6)	12 (23.1)	4 (30.8)	
Panuveitis	32 (49.2)	28 (53.8)	4 (30.8)	
**Anterior segment findings, n (%)**				
Conjunctival hyperemia	16 (24.6)	15 (28.8)	1 (7.7)	0.159
Anterior chamber reaction	52 (80.0)	45 (86.5)	7 (53.8)	0.016
Keratic precipitates	30 (46.2)	25 (48.1)	5 (38.5)	0.757
Posterior synechiae	6 (9.2)	6 (11.5)	0 (0)	0.335
**Posterior segment findings, n (%)**				
Necrotizing retinitis	11 (16.9)	10 (19.2)	1 (7.7)	0.439
Multifocal inflammation of the retina/RPE	17 (26.2)	14 (26.9)	3 (23.1)	1
Retinal vasculitis	25 (38.5)	23(44.2)	2 (15.4)	0.065
Optic disc edema/hyperemia	32 (49.2)	25 (48.1)	7 (53.9)	0.076
Acute syphilitic posterior placoid chorioretinopathy	1 (1.5)	1 (1.9)	0 (0.0)	1
**Anterior chamber cells, n (%)**				
Grade 0	13 (20.0)	7 (13.5)	6 (46.2)	0.057
0.5 +	9 (13.8)	7 (13.5)	2 (15.4)	
1 +	20 (30.8)	18 (34.5)	2 (15.4)	
≥ 2 +	23 (35.4)	20 (38.5)	3 (23.0)	
**Vitreous haze, n (%)**				
Grade 0	15 (28.8)	12 (26.7)	3 (42.8)	0.347
0.5 +	15 (28.8)	15 (33.3)	0 (0)	
1 +	11 (21.2)	9 (20)	2 (28.6)	
≥ 2 +	11 (21.2)	9 (20)	2 (28.6)	
Missing	13	7	6	
**Ocular complication at presentation, n (%)**				
Ocular hypertension	2 (3.1)	1 (1.9)	1 (7.7)	0.096
Vitreous hemorrhage	2 (3.1)	0 (0)	2 (15.4)	
Cystoid macular edema	4 (6.2)	4 (7.7)	0 (0)	
Serous retinal detachment	4 (6.2)	4 (7.7)	0 (0)	
Branch retinal vein occlusion	1(1.5)	0 (0)	1 (7.7)	
Hypotony	1(1.5)	1(1.9)	0 (0)	
**CSF abnormalities, n (%)**^**a**^				
Yes	20 (83.3)	18 (94.7)	2 (40.0)	0.018
No	4 (16.7)	1 (5.3)	3 (60.0)	
Mean final logMAR BCVA(SD)	0.31 (0.60)	0.35 (0.66)	0.16 (0.16)	0.342
Median final logMAR BCVA (IQR)	0.13 (0.06, 0.24)	0.14 (0.04, 0.3)	0.12 (0.08, 0.19)	0.654
**Final BCVA, n (%)**^**b**^				
≥ 20/25	39 (62.9)	30 (60.0)	9 (75.0)	0.688
20/32–20/40	12 (19.3)	10 (20.0)	2 (16.7)	
20/50–20/160	7 (11.3)	6 (12.0)	1 (8.3)	
≤ 20/200	4 (6.5)	4 (8.0)	0 (0)	

^a^Calculated in 24 patients who underwent lumbar puncture.

^b^Calculated in 62 eyes as three eyes were lost to follow-up before receiving antibiotic treatment.

BCVA: best corrected visual acuity; SD: standard deviation; IOP: intraocular pressure; RPE: retinal pigment epithelium.

In comparing patients with and without HIV coinfection, the eyes of HIV-infected patients had an increased likelihood of worse VA on presentation (Snellen equivalent = 20/250 vs. 20/57, *p* = 0.003) and having an anterior chamber reaction (87 vs 54%, *p* = 0.016) compared to those of HIV-uninfected patients. There were no significant differences in the anatomic location of uveitis, proportion of any posterior segment finding, severity of cellular grading, and ocular complications at presentation between the two groups.

Follow-up data on serum nontreponemal test titers were available in 14 patients (39%). Of them, 13 achieved a four-fold decline in titer within one year of antibiotic treatment. However, one patient had persistently elevated titers. For this patient, repeat CSF examination was performed and retreatment with a month-long course of doxycycline was initiated.

### VA outcomes after treatment with systemic antibiotics

**Figure 2:**
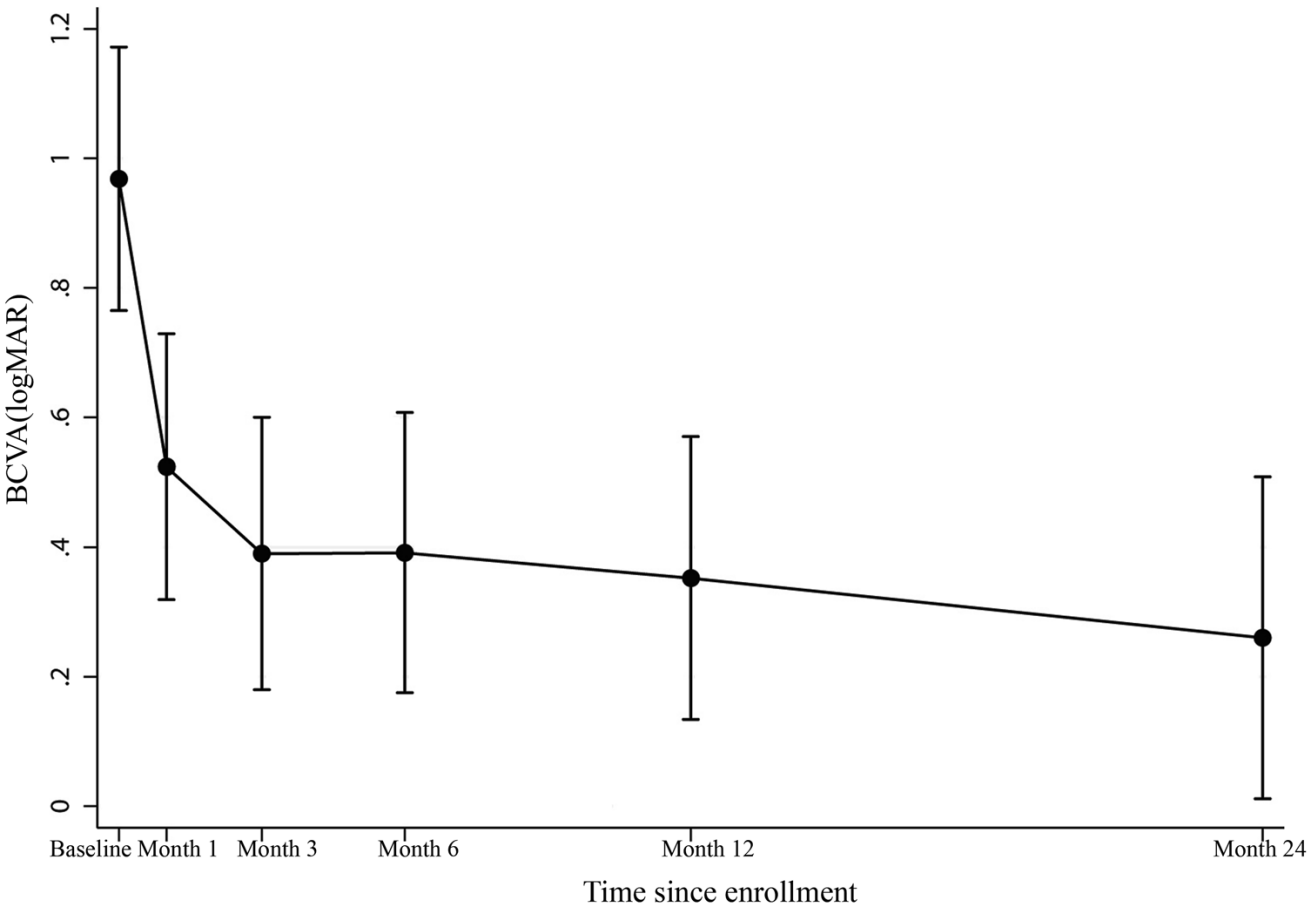
Estimates of mean best corrected visual acuity (BCVA) over two years after presentation produced by a linear mixed model. Vertical lines indicate the 95% confidence interval.

The VA observations over the first two years after presentation are presented in Table 3. Overall, 51 eyes (82%) demonstrated an improvement in VA of ≥ 1 line by the time of the last follow-up. The modeled mean logMAR VA which derived from mixed effect random-intercept linear regression was 0.97 (95% confidence interval [CI], 0.76–1.17) (Snellen equivalent = 20/187) at presentation. This significantly improved to 0.52 (95% CI, 0.32–0.73) (Snellen equivalent = 20/66) at 1 month (*p* < 0.001), and to 0.39 (95% CI, 0.18–0.60) (Snellen equivalent = 20/49) at 3 months (*p* < 0.001). After 3 months, the BCVA remained relatively stable throughout the 2-year follow-up period (*p* > 0.05 at every subsequent interval) (Fig. 2).

**Table 3:** Visual acuity outcomes after treatment with systemic antibiotics.

	Baseline	Month 1	Month 3	Month 6	Month 12	Month 24
No. of eyes, n	65	62	51	41	38	18
**BCVA range, n (%)**						
≥ 20/25	8 (12.3)	29 (46.8)	27 (52.9)	25 (61.0)	25 (65.8)	8 (44.4)
20/32 to 20/40	15 (23.1)	9 (14.5)	8 (15.8)	8 (19.5)	4 (10.5)	3 (16.7)
20/50 to 20/160	18 (27.7)	15 (24.2)	9 (17.6)	3 (7.3)	5 (13.2)	3 (16.7)
≤ 20/200	24 (36.9)	9 (14.5)	7 (13.7)	5 (12.2)	4 (10.5)	4 (22.2)
Mean logMAR VA (95%CI)^a^	0.97(0.76, 1.17)	0.52 (0.32, 0.73)	0.39 (0.18, 0.60)	0.39 (0.18, 0.61)	0.35 (0.13, 0.57)	0.26 (0.01, 0.51)
Snellen equivalent	20/187 (20/115, 20/295)	20/66 (20/42, 20/107)	20/49 (20/30, 20/80)	20/49 (20/30, 20/81)	20/45 (20/28, 20/76)	20/36 (20/20, 20/65)
P value^b^		< 0.001	< 0.001	< 0.001	< 0.001	< 0.001

*BCVA*: Best-corrected visual acuity; *logMAR*: log of the minimum angle of the resolution.

^a^Derived from mixed effect random-intercept linear regression model.

^b^Compared to baseline.

**Figure 3:**
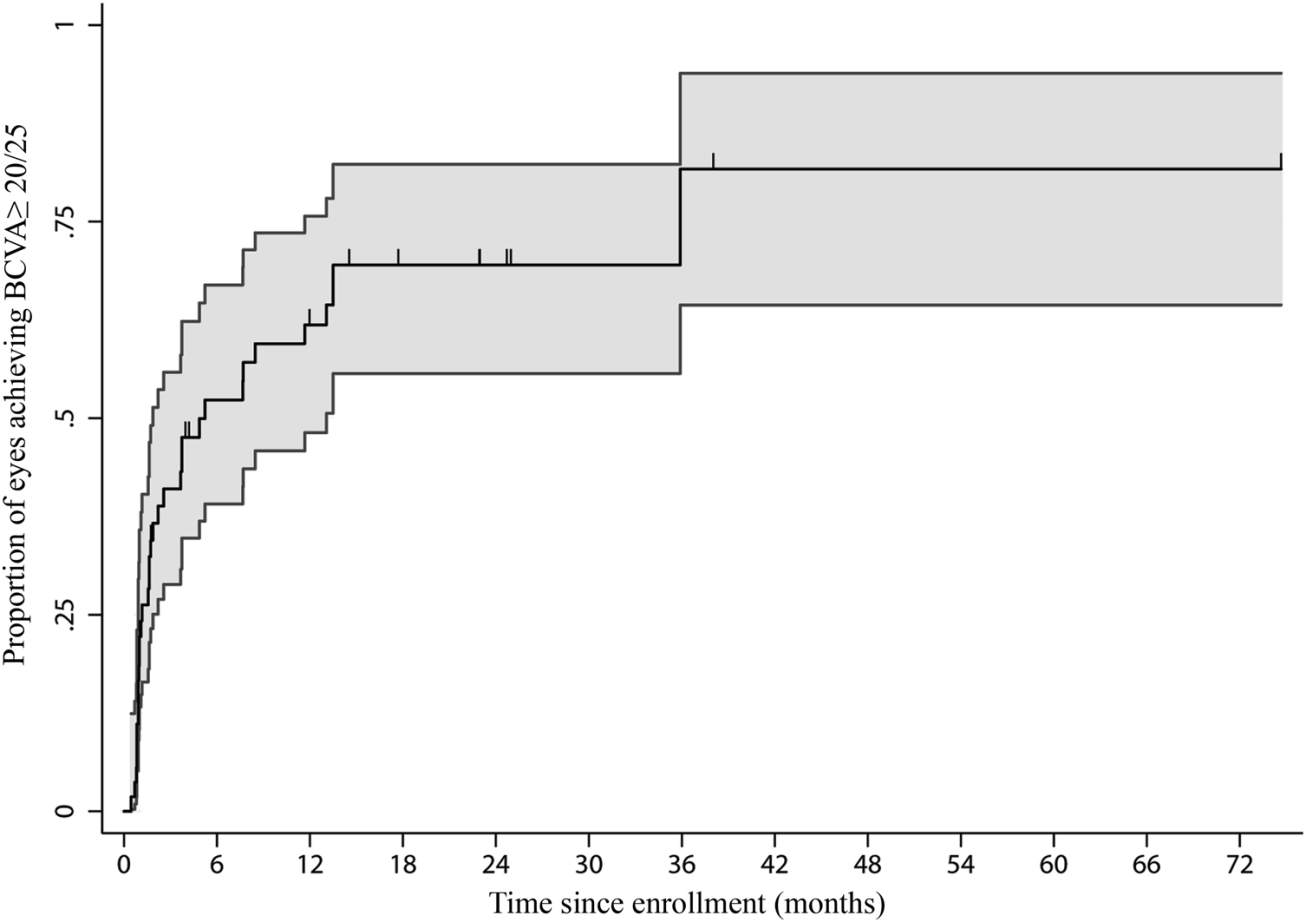
Kaplan–Meier estimates of the overall cumulative probability of best corrected visual acuity (BCVA) improvement to ≥ 20/25 among a total of 54 eyes with baseline BCVA of < 20/25. Shaded areas denote 95% confidence intervals.

The incidence of VA improvement to ≥ 20/25 following antibiotic treatment and the relationships between characteristics are summarized in Table 4. The VA values during follow-up were available in 62 of 65 eyes. Among the 62 eyes, 54 had a VA of < 20/25 at first presentation and were then included in the incidence analyses. These 54 eyes had been followed over 561.9 EMs; among these, 35 eyes achieved a BCVA of ≥ 20/25 (incidence rate 0.06/EM; 95% CI, 0.05–0.09). The cumulative incidence of achieving BCVA ≥ 20/25 is shown in Fig. 3. Overall, BCVA improvement to ≥ 20/25 was achieved in 70% (95% CI, 55.7–82.3) of eyes within the 2-year follow-up time frame. The median time to achieving VA ≥ 20/25 was 5.2 months (95% CI, 3.91–6.70).

**Table 4:** Incidence of and predictive factors for BCVA improvement to ≥ 20/25 in eyes of patients with syphilitic uveitis following antibiotic therapy.

	*n/N*^a^	Percentage of BCVA improvement to ≥ 20/25 following therapy by month							
		Rate/EM (95%CI)^b^	1mo (%)	6mo (%)	12mo (%)	24mo (%)	*P* value (Log-rank)	Crude HR (95% CI)^c^	*P* value (LR test)^d^
Overall rate	35/54	0.062 (0.045, 0.086)	22.2	52.3	61.9	69.5	–	–	–
**Age (years)**									
≤ 36	20/32	0.055 (0.036, 0.084)	15.6	55.2	68.6	68.6	0.925	1	
> 36	15/22	0.076 (0.047, 0.124)	31.8	50.4	55.4	70.3		1.03 (0.42, 2.52)	0.942
**Sex**									
Male	33/52	0.059 (0.042, 0.082)	19.2	50.5	60.4	68.3	0.026	1	
Female	2/2	1.000 (1, 1)	100	–	–	–		4.69 (2.28, 9.65)	< 0.001
**MSM**									
No	4/9	0.026 (0.010, 0.069)	0	14.3	14.3	28.6	0.03	1	
Yes	23/32	0.108 (0.073, 0.158)	18.8	60.7	73.8	82.5		3.10 (1.23, 7.81)	0.016
**Duration of ocular symptoms prior to presentation**									
≤ 4 weeks	11/11	0.305 (0.186, 0.499)	18.2	81.8^e^	–	–	0.006	1	
> 4 weeks	24/43	0.046 (0.031, 0.067)	23.3	43	49.3	59.4		0.36 (0.17, 0.75)	0.006
**Serum nontreponemal titer**									
≤ 1:64	23/32	0.092 (0.062, 0.135)	25	55.5	67.6	75.7	0.234	1	
> 1:64	12/22	0.039 (0.022, 0.067)	18.2	47.6	53.4	60		0.66 (0.27, 1.62)	0.363
**HIV infection**									
Yes	27/45	0.052 (0.036, 0.074)	15.6	47.1	58.9	65.2	0.016	1	
No	8/9	0.209 (0.113, 0.387)	55.6	77.8	77.8	–		2.58 (0.79, 8.47)	0.118
**Receiving ART at presentation**^**f**^									
No	25/37	0.078 (0.053, 0.114)	13.5	50.7	64.8	72.6	0.081	1	
Yes	2/8	0.010 (0.002, 0.039)	25	25	25	25		0.31 (0.05, 1.96)	0.212
**CD4 counts at presentation**^**f**^									
≤ 200 cells/µL	11/22	0.040 (0.023, 0.072)	13.6	36.1	47.7	47.7	0.078	1	
> 200 cells/µL	16/21	0.065 (0.040, 0.104)	19.1	59.4	71	82.6		1.95 (0.78, 4.86)	0.152
**Receiving adjunctive prednisolone after antibiotic**s									
No	20/33	0.071 (0.046, 0.108)	27.3	54.7	62.9	62.9	0.789	1	
Yes	12/15	0.097 (0.057, 0.167)	0	47.5	62.5	85		1.10 (0.51, 2.41)	0.804
**Presence of neurosyphilis**									
No	5/5	0.949 (0.779, 1.156)	80	–	–	–	< 0.001	1	
Yes	19/32	0.071 (0.046, 0.109)	15.6	48.8	56.7	69.7		0.15 (0.05, 0.48)	0.001
**Baseline BCVA**									
< 20/25 to 20/40	11/15	0.194 (0.114, 0.330)	33.3	68.3	89.4	–	< 0.001	1	
< 20/40 to > 20/200	14/17	0.277 (0.177, 0.432)	29.4	80.4	90.2	–		1.08 (0.48, 2.43)	0.848
20/200 or worse	10/22	0.022 (0.012, 0.041)	9.1	23.7	28.8	39.8		0.18 (0.07, 0.47)	0.001
Trend								0.45 (0.30, 0.69)	< 0.001
**Anatomic location of uveitis**									
Anterior uveitis	0/0	–	–	–	–	–		–	
Intermediate uveitis	9/12	0.631 (0.424, 0.938)	58.4	–	–	–	< 0.001	1	
Posterior uveitis	9/14	0.045 (0.024, 0.085)	21.4	49.1	59.3	79.6		0.23 (0.06, 0.84)	0.027
Panuveitis	17/28	0.049 (0.031, 0.078)	7.1	40.8	52.6	57		0.16 (0.06, 0.44)	< 0.001
**Anterior chamber cells grading**									
0 to 2 +	26/43	0.051 (0.036, 0.075)	18.6	46.7	56.1	65.5	0.064	1	
3 + to 4 +	9/11	0.159 (0.087, 0.290)	36.4	72.7	81.8	81.8		2.03 (0.87, 4.73)	0.1
**Vitreous haze grading**									
0 to 1 +	24/36	0.075 (0.052, 0.111)	19.4	61.1	72.8	80.6	0.275	1	
2 + to 4 +	4/8	0.046 (0.017, 0.119)	25	37.5	53.1	53.1		0.56 (0.15, 2.09)	0.391
**Antibiotic regimen**									
Intravenous Penicillin G	31/50	0.064 (0.045, 0.091)	22	55	62.9	71.5	0.999	1	
Other^g^	4/4	0.050 (0.019, 0.129)	25	25	50	50		0.99 (0.34, 2.97)	0.999
**Presence of any ocular complication at presentation**									
No	29/42	0.102 (0.072,0.144)	26.2	55.7	69.3	77	0.062	1	
Yes	6/12	0.022 (0.010, 0.048)	8.3	41.7	41.7	50		0.44 (0.14, 1.42)	0.17

*ART*: Antiretroviral therapy; *BCVA*: Best corrected visual acuity; *CI*: Confidence interval; *CSF*: Cerebrospinal fluid; *EM*: Eye-months; *HIV*: Human immunodeficiency virus; *MSM*: Men who have sex with men; mo: month.

^a^n/N indicates number of events/number of eyes at risk.

^b^Incidence rate is number of events divided by the total eye-months at risk.

^c^Crude HR = hazard ratio for univariate Cox proportional model.

^d^Likelihood ratio test.

^e^The events BCVA of ≥ 20/25 were achieved at 36 months after therapy.

^f^Calculated in eyes of HIV- infected patients only.

^g^Others included ceftriaxone and azithromycin.

Multivariate Cox regression analyses based on the DAG model were performed to identify significant predictors associated with VA improvement to ≥ 20/25 (Table 5). Significant predictors included: female (adjusted hazard ratio [aHR] = 10.7; 95% CI, 3.5–32.7, *p* < 0.001); MSM (aHR = 2.8; 95% CI, 1.2–6.9, *p* = 0.023); duration of ocular symptoms prior to treatment of ≤ 4 weeks (aHR = 3.4; 95% CI, 1.3–9.0, *p* = 0.012); eyes of HIV-uninfected patients as opposed to those co-infected with HIV who had CD4 counts of ≤ 200 cells/μL (aHR = 8.2; 95% CI, 2.5–26.5, *p* < 0.001) and of > 200 cells/μL (aHR = 5.7; 95% CI, 1.4–23.1, *p* = 0.015); absence of neurosyphilis (aHR = 6.5; 95% CI, 1.1–38.0, *p* = 0.037); and eyes with MVL at presentation as opposed to SVL (aHR 5.0; 95% CI, 1.7–14.2, *p* = 0.003). In addition, eyes with intermediate uveitis, as opposed to panuveitis (aHR = 11.5; 95% CI, 1.7–79.8, *p* = 0.013) were significantly associated with achieving a VA of ≥ 20/25.

**Table 5:** Multivariate analyses by robust estimation of predictive factors associated with achieving BCVA of ≥ 20/25 following antibiotic therapy.

Characteristics	Minimally sufficient adjustment set	Exposure variable: level	BCVA of ≥ 20/25	
			Adjusted hazard ratio (95% CI)	*P* value (Wald test)
Demographics	No adjustment			
Age (years)		≤ 36	1	
		> 36	0.99 (0.41, 2.42)	0.990
Sex		Male	1	
		Female	10.69 (3.50, 32.68)	< 0.001
MSM		No	1	
		Yes	2.83 (1.16, 6.93)	0.023
Duration of ocular symptoms prior to presentation (weeks)	Demographics	> 4 weeks	1	
		≤ 4 weeks	3.43 (1.31, 8.99)	0.012
HIV status	Demographics			
HIV infection by CD4 count (cells/µL)		HIV + and CD4 ≤ 200	1^a^	
		HIV + and CD4 > 200	1.43 (0.55, 3.69)^a,b^	0.462
		HIV-uninfected	8.15 (2.51, 26.47)^c^	< 0.001
Receiving ART at presentation		No	1	
		Yes	0.21 (0.02, 1.81)	0.154
Severity of systemic syphilis	HIV status			
Presence of neurosyphilis		Yes	1	
		No	6.51 (1.11, 38.02)	0.037
Baseline BCVA	Clinical characteristics	20/200 or worse	1 ^a^	
	Presence of any ocular complication at presentation	< 20/40 to > 20/200	4.99 (1.75, 14.22) ^b^	0.003
	Duration of ocular symptoms prior to presentation	< 20/25 to 20/40	2.27 (0.47, 11.00) ^a,b^	0.310
	HIV status	Trend	1.65 (0.87, 3.14)	0.128
Clinical characteristics	Duration of ocular symptoms prior to presentation	Panuveitis	1^a^	
Anatomic location of uveitis	HIV status	Posterior uveitis	1.44 (0.43, 4.80) ^a,b^	0.549
	Severity of systemic syphilis	Intermediate uveitis	11.51 (1.66, 79.80) ^b^	0.013
Systemic antibiotic regimens	Clinical characteristics	IV penicillin G	1	
	Baseline BCVA	Others^#^	3.95 (0.41, 38.03)	0.235
	Severity of systemic syphilis			
Presence of any ocular complication at presentation	Demographics	No	1	
	Duration of ocular symptoms prior to presentation	Yes	0.26 (0.03, 2.56)	0.247
	HIV status			
Adjunctive systemic steroids after antibiotic therapy	Baseline BCVA	No	1	
	Clinical characteristics	Yes	0.78 (0.34, 1.80)	0.560
	Presence of any ocular complication at presentation			

The abc values in columns not having a superscript in common differ significantly (P < 0.05).

^#^Others included ceftriaxone and azithromycin.

ART: antiretroviral therapy; BCVA: best corrected visual acuity; HIV: human immunodeficiency virus;

IV: intravenous; MSM: men who have sex with men.

Among the 35 eyes that achieved a VA improvement to ≥ 20/25, six eyes worsened to < 20/25 by the last visit. Of these, two worsened to 20/40 and four worsened to 20/32. Causes included cataracts (four eyes), mild ERM (one eye), and mild CME (one eye).

## Discussion

The present study reports on the clinical and laboratory findings, and VA outcomes of patients with syphilitic uveitis, with and without HIV coinfection, following systemic antibiotic therapy. We observed that most patients were young MSMs who had HIV co- infection. The majority of patients had high initial serum nontreponemal titers, suggesting that they were likely in earlier disease stages when they experienced ocular symptoms and presented at our clinic, rather than later stages, e.g., late latent or tertiary syphilis^2,4,25^. Following antibiotic therapy, a substantial degree of VA improvement was achieved. Additionally, approximately 70% of eyes were able to achieve a VA ≥ 20/25 by 2 years after presentation. Receiving early antibiotic treatment, absence of HIV coinfection, absence of neurosyphilis, better VA at presentation, and intermediate uveitis as opposed to panuveitis were predictive of achieving a VA of ≥ 20/25.

The demographic profile of our patients is consistent with the epidemiologic findings of systemic and ocular syphilis from North America and Western Europe, in which MSM with HIV coinfection represented the majority of the cohort^5^. Furthermore, our data adds to the existing information regarding similar epidemiologic trends of systemic syphilis in Thailand that have been rising, significantly, since 2005 among Thai MSM with HIV infection^26–28^. In our study, we found a significantly high rate of HIV coinfection (80%). Furthermore, most patients were newly diagnosed with HIV infection after the syphilis diagnosis. These results support the US Centers for Disease Control and Prevention’s recommendation of conducting HIV testing in all patients diagnosed with ocular syphilis^29^. Systemic features of syphilis were observed in roughly one-third of our patients; the proportion found in our study was in the lower range compared to those of earlier studies, which have reported rates ranging from 21 to 70% among patients with syphilitic uveitis^10,14,30^. This low rate, in particular the rate of genital chancre, could be due to the fact that the data we obtained were mostly based on patient self-reports. A thorough physical examination to document the existence of either active or healed mucocutaneous lesions might have not been performed in all cases.

We found that posterior and panuveitis were the main uveitis presentations. Necrotizing retinitis was predominantly observed in our HIV-infected patients. This observation is consistent with the characteristics of cases that were collected and analyzed in the SUN database to develop classification criteria for syphilitic uveitis. In that study, necrotizing retinitis was observed only in patients with HIV infection and the authors proposed that this retinitis feature may be a variant associated with a compromised immune response^21^. The two distinct retinochoroidal appearances described as a specific presentation of ocular syphilis, which included opacified retinitis with superficial retinal precipitates and acute syphilitic posterior placoid chorioretinopathy (ASPPC)^31–33^, were found in three eyes and one eye in our cohort, respectively (see Supplementary Fig. S1-2). We acknowledge that the prevalence of ASPPC in our study could have been underestimated, as previous studies have reported that the rate of ASPPC varied from 10 to 25% among patients with syphilitic uveitis^10,17,21^. This underestimation may be explained by the fact that the natural course of ASPPC evolves over time. In addition, the placoid lesions might be difficult to recognize on fundus examination, especially early in the disease course^34^. The incorporation of multimodal imaging such as fundus autofluorescence, OCT, or angiogram could be greatly useful for identifying subclinical areas of such lesions. However, they were not systematically performed in all our patients.

The VA results suggest that visual outcomes of syphilitic uveitis are favorable in response to antibiotic treatment. The modeled VA derived from the mixed-effects random-intercept linear regression, which accounted for the propensity of patients with good VA who stop coming to follow-up appointments over time, demonstrated that VA tended to improve shortly after receiving antibiotics and was well maintained at approximately 20/49 for up to two years after presentation. Furthermore, an increased likelihood of recovery of VA to ≥ 20/25 was observed among eyes receiving early antibiotic treatment and eyes with a better presenting VA, which is consistent with previous studies^12,13,17,35^. In contrast, eyes with a presenting VA of ≤ 20/200 were less likely to regain VA, which was attributed to the presence of retinal scarring due to extensive retinitis lesions, RD, and recalcitrant CME. The anatomic location of uveitis was predictive of VA improvement. As expected, eyes with intermediate uveitis, wherein the inflammation was primarily localized in the vitreous cavity, were more likely to regain VA compared to those with panuveitis in which the inflammation extended to involve the retina or choroid.

Another notable finding is the presence of HIV coinfection, which decreased the likelihood of VA improvement, and this effect appeared to correlate with the degree of immunosuppression as evidenced by CD4 counts. However, a significant difference was not observed between those HIV patients with CD4 counts ≤ 200 and > 200 cells/µL. The effect of subsequent use of ART and immune recovery in those patients with initially low CD4 count, may assist in their VA restoration, and account for the non-significant result observed. Among 22 HIV-infected patients who were not taking ART at presentation, all subsequently received ART during the follow-up. Some previous studies did not establish HIV coinfection as a predictor of visual outcome, in contrast to the present study^1,8,10,32,35^. The discrepancy may be due to inconsistencies in the assessment method of VA outcome and variability in the level of immunosuppression among HIV-infected individuals in each study. The more advanced and untreated HIV infection present in our patients might be responsible for the poorer visual prognosis. Approximately half of the HIV-infected patients had baseline CD4 counts of ≤ 200 cells/µL. In HIV-infected patients, reduced opsonic activity of macrophages and decreased clearance of *T. pallidum* from local sites of infection were observed^36^. Hence, the impairment of spirochete clearance from ocular tissue may prolong and aggravate the intraocular infectious process and decrease the likelihood of VA recovery in the HIV-infected population, as demonstrated in this study.

The results of this study show a high rate of neurosyphilis (84%) that was almost exclusively observed in HIV-infected patients. The prevalence of neurosyphilis reported earlier varied between 25 and 83%, depending on case selection and diagnostic criteria applied in each study^37,38^. The high proportion of HIV-infected patients with low CD4 counts and the high initial serum nontreponemal test titers in our patients are likely to be responsible for the high neurosyphilis rate observed^39,40^. It is notable that the presence of concomitant neurosyphilis also decreased the likelihood of VA recovery after taking into account HIV status. The results of the analysis did not change when four patients diagnosed as neurosyphilis with only elevated CSF protein concentrations were excluded. To date, limited evidence exists on the relationship between neurosyphilis and visual outcomes in ocular syphilis cases. We hypothesized that neurosyphilis might result in an occlusive/ischemic microvasculopathy involving the optic nerve and the associated visual pathway and contribute to the poorer recovery of VA in these patients. Our data suggest that performing LP to establish whether neurosyphilis is present may add a benefit in providing patients’ visual prognoses. This finding may be worthy of consideration in light of the 2021 updated guideline by the US Centers for Disease Control and Prevention which states that CSF examination is not always needed before treatment for persons with ocular syphilis if there is no evidence of cranial nerve dysfunction or other neurologic abnormalities, as the result of CSF analyses does not alter the initial management and follow-up^29^.

Our study did not observe the significant difference on VA recovery between those who did or did not receive adjunctive oral prednisolone following systemic antibiotic therapy. However, this result should be interpreted with caution owing to the non-standardized doses and duration of prednisolone prescribed in our patients. Further, the decision to initiate systemic steroids was typically based on the severity of intraocular inflammation. Patients with more severe inflammation were more likely to be prescribed prednisolone which could have introduced indication bias. Randomized controlled trial would be the most suitable study design to explore the benefit of adjunctive systemic steroid treatment.

Our results demonstrated that the female patient had a greater probability of achieving VA recovery than the male patients. It is important to note that this result was based on only one female participant. Therefore, no conclusion can be drawn on this association. Further studies with a larger number of female patients are required to provide evidence and confirm such associations. We observed an increased likelihood of achieving VA recovery among the MSM compared to the non-MSM population. This finding may be attributed to a shorter median duration of ocular symptoms prior to treatment (4.3 vs. 17.1 weeks, *p* = 0.64, Mann–Whitney U test), better median presenting VA (0.74 logMAR vs. 1.3 logMAR, *p* = 0.46, Mann–Whitney U test), and higher median baseline CD4 counts among those who were co-infected with HIV (329 vs 142 cells/µL, *p* = 0.057, Mann–Whitney U test) in the MSM group compared to the non-MSM group.

This study has several limitations. First, the retrospective nature and relatively small sample, mainly consisting of men living with HIV who presented with early syphilis, used in the study may limit its generalizability. Second, other predictive factors of VA improvement may be unrecognized. These include HIV viral load and multimodal imaging findings, such as OCT imaging and angiography, which were not included due to insufficient data. Third, we only used VA as the measure of visual function, which does not completely represent all aspects of vision-related to functioning. Fourth, there may be referral bias since the study was conducted at a tertiary center. However, few, if any, other hospitals in our region comprehensively manage patients with uveitis involving the posterior segment. Hence, we believe that our data are fairly representative of uveitis caused by syphilis in southern Thailand and are not limited to only a tertiary setting. The results of the present study could contribute to the data on ocular syphilis from developing countries located in the South East Asia region where HIV and STIs are currently a high burden.

In summary, men with HIV coinfection represented the majority of our patients with syphilitic uveitis. These demographic characteristics were consistent with the current epidemiologic data of syphilis resurgence in western countries. Bilateral uveitis with posterior segment involvement was a common presentation. Substantial degree of VA improvement was achieved in most eyes with a short course of antibiotic therapy, this finding highlights the importance of accurately diagnosing syphilis. Delayed treatment with poor VA at presentation, presence of HIV coinfection, and concomitant neurosyphilis decreased the likelihood of VA restoration. In the era of rapidly evolving multimodal ocular imaging, future studies that focus on the combination of different imaging modalities are warranted for identification of potential biomarkers to serve as reliable predictors of disease severity and functional outcomes post-therapy.

## Supplementary Information

Supplementary Information.

## Data Availability

The datasets generated during and/or analyzed during the current study are available from the corresponding author on reasonable request.

## References

[1] Mathew RG, Goh BT, Westcott MC (2014). British ocular syphilis study (BOSS): 2-year national surveillance study of intraocular inflammation secondary to ocular syphilis. Invest. Ophthalmol. Vis. Sci..

[2] Furtado JM (2022). Ocular syphilis. Surv. Ophthalmol..

[3] Rowley J (2019). Chlamydia, gonorrhoea, trichomoniasis and syphilis: Global prevalence and incidence estimates, 2016. Bull. World Health Organ..

[4] Hook EW (2017). Syphilis. Lancet.

[5] Abara W. E, Hess, K. L, Neblett Fanfair, R., Bernstein, K. T., Paz-Bailey, G. Syphilis trends among men who have sex with men in the United States and Western Europe: A systematic review of trend studies published between 2004 and 2015. *PLOS ONE*. **11**, e0159309; 10.1371/journal.pone.0159309 (2016). 10.1371/journal.pone.0159309 PMC4957774 27447943

[6] Oliver SE (2016). Ocular syphilis–eight jurisdictions, United States, 2014–2015. MMWR Morb. Mortal Wkly Rep..

[7] Oliver GF (2019). Current ophthalmology practice patterns for syphilitic uveitis. Br. J. Ophthalmol..

[8] Furtado, J. M. *et al*. Clinical manifestations and ophthalmic outcomes of ocular syphilis at a time of re-emergence of the systemic infection. *Sci Rep*. **8**, 12071; 10.1038/s41598-018-30559-7 (2018). 10.1038/s41598-018-30559-7 PMC6089995 30104765

[9] Northey LC, Skalicky SE, Gurbaxani A, McCluskey PJ (2015). Syphilitic uveitis and optic neuritis in Sydney Australia. Br. J. Ophthalmol..

[10] Fonollosa A (2016). Clinical manifestations and outcomes of syphilis-associated uveitis in northern Spain. Ocul. Immunol. Inflamm..

[11] Mathew D, Smit D (2021). Clinical and laboratory characteristics of ocular syphilis and neurosyphilis among individuals with and without HIV infection. Br. J. Ophthalmol..

[12] Queiroz, R. P. *et al*. The ghost of the great imitator: Prognostic factors for poor outcome in syphilitic uveitis. *J. Ophthal. Inflam. Infect*. **9**, 2; 10.1186/s12348-019-0169-8 (2019). 10.1186/s12348-019-0169-8 PMC6338615 30659387

[13] Moradi A (2015). Clinical features and incidence rates of ocular complications in patients with ocular syphilis. Am. J. Ophthalmol..

[14] Moramarco A (2020). Clinical features of ocular syphilis: A retrospective clinical study in an Italian referral centre. Semin. Ophthalmol..

[15] Hoogewoud F (2017). Prognostic factors in syphilitic uveitis. Ophthalmology.

[16] Zhang, T., Zhu, Y., Xu, G. Clinical features and treatments of syphilitic uveitis: A systematic review and meta-analysis. *J. Ophthalmol*. **2017**, 6594849; 10.1155/2017/6594849 (2017). 10.1155/2017/6594849 PMC5511639 28751982

[17] Tsuboi M (2016). Prognosis of ocular syphilis in patients infected with HIV in the antiretroviral therapy era. Sex. Transm. Infect..

[18] Yang P, Zhang N, Li F, Chen Y, Kijlstra A (2012). Ocular manifestations of syphilitic uveitis in Chinese patients. Retina.

[19] Thailand Department of Disease Control. Syphilis. *DDC Watch* https://ddc.moph.go.th/uploads/publish/771820191010055224.pdf (2019).

[20] US Public Health Service (1978). ICD-9-CM, clinical modification of ICD-9 for Use in the USA.

[21] Standardization of uveitis nomenclature (SUN) working group. Classification criteria for syphilitic uveitis. *Am. J. Ophthalmol*. **228**,182–191 (2021). 10.1016/j.ajo.2021.10.020 34715075

[22] Jabs, D. A., Nussenblatt, R. B., Rosenbaum, J. T., Standardization of Uveitis Nomenclature (SUN) Working Group. Standardization of uveitis nomenclature for reporting clinical data. Results of the first international workshop. *Am. J. Ophthalmol*.**140**, 509–516 (2005). 10.1016/j.ajo.2005.03.057 PMC8935739 16196117

[23] Marra, C. M., Maxwell, C. L., Collier, A. C., Robertson, K. R., Imrie, A. Interpreting cerebrospinal fluid pleocytosis in HIV in the era of potent antiretroviral therapy. *BMC Infect Dis*. **7**: 37; 10.1186/1471-2334-7-37 (2007). 10.1186/1471-2334-7-37 PMC1871592 17475004

[24] Dunaway SB, Maxwell CL, Tantalo LC, Sahi SK, Marra CM (2020). Neurosyphilis treatment outcomes after intravenous penicillin G versus intramuscular procaine penicillin plus oral probenecid. Clin. Infect. Dis..

[25] Hook EW, Marra CM (1992). Acquired syphilis in adults. N. Engl. J. Med..

[26] Muccini C (2021). Brief report: Syphilis incidence and effect on viral load, CD4, and CD4/CD8 ratio in a Thai cohort of predominantly men who have sex with men living with HIV. J. Acquir. Immune. Defic. Syndr..

[27] Holtz TH (2019). Why we need pre-exposure prophylaxis: Incident HIV and syphilis among men, and transgender women, who have sex with men, Bangkok, Thailand, 2005–2015. Int. J. STD AIDS..

[28] Centers for Disease Control and Prevention (CDC). HIV and syphilis infection among men who have sex with men—Bangkok, Thailand, 2005–2011. *MMWR Morb. Mortal. Wkly Rep*. **62**, 518–520 (2013). PMC4604950 23803960

[29] Workowski KA (2021). Sexually transmitted infections treatment guidelines, 2021. MMWR Recomm. Rep..

[30] Balaskas K, Sergentanis TN, Giulieri S, Guex-Crosier Y (2011). Analysis of significant factors influencing visual acuity in ocular syphilis. Br. J. Ophthalmol..

[31] Fu EX (2010). Superficial retinal precipitates in patients with syphilitic retinitis. Retina.

[32] Eandi CM (2012). Acute syphilitic posterior placoid chorioretinitis: Report of a case series and comprehensive review of the literature. Retina.

[33] Gorovoy IR, Desai S (2013). Syphilitic posterior placoid chorioretinitis. Sex. Transm. Dis..

[34] Pichi F, Neri P (2020). Multimodal imaging patterns of posterior syphilitic uveitis: A review of the literature, laboratory evaluation and treatment. Int Ophthalmol..

[35] Bollemeijer JG (2016). Clinical manifestations and outcome of syphilitic uveitis. Invest. Ophthalmol. Vis. Sci..

[36] Marra CM, Tantalo LC, Sahi SK, Dunaway SB, Lukehart SA (2016). Reduced Treponema pallidum-specific opsonic antibody activity in HIV-infected patients with syphilis. J. Infect. Dis..

[37] Amaratunge BC, Camuglia JE, Hall AJ (2010). Syphilitic uveitis: A review of clinical manifestations and treatment outcomes of syphilitic uveitis in human immunodeficiency virus-positive and negative patients. Clin. Exp. Ophthalmol..

[38] Queiroz RP, Smit DP, Peters RPH, Vasconcelos-Santos DV (2020). Double trouble: Challenges in the diagnosis and management of ocular syphilis in HIV-infected individuals. Ocul. Immunol. Inflamm..

[39] Marra CM, Maxwell CL, Smith SL (2004). Cerebrospinal fluid abnormalities in patients with syphilis: Association with clinical and laboratory features. J. Infect. Dis..

[40] Hobbs E, Vera JH, Marks M, Barritt AW, Ridha BH, Lawrence D (2018). Neurosyphilis in patients with HIV. Pract. Neurol..

