# Supplementary material for: Clinical features and incidence of visual improvement following systemic antibiotic treatment in patients with syphilitic uveitis
Source: Sci Rep. 2022 Jul 22;12:12553. doi: 10.1038/s41598-022-16780-5 (PMC9307809; doi:10.1038/s41598-022-16780-5)
Supplement: Supplementary file 1 — Supplementary Information. [file 41598_2022_16780_MOESM1_ESM.docx]

**
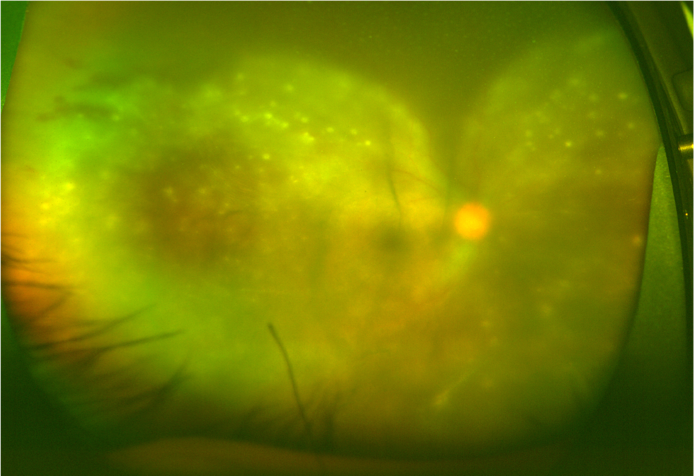
**

**Figure S1.** Wide-field fundus photograph demonstrating the feature of opacified retinitis with multiple superficial retinal precipitates.

**
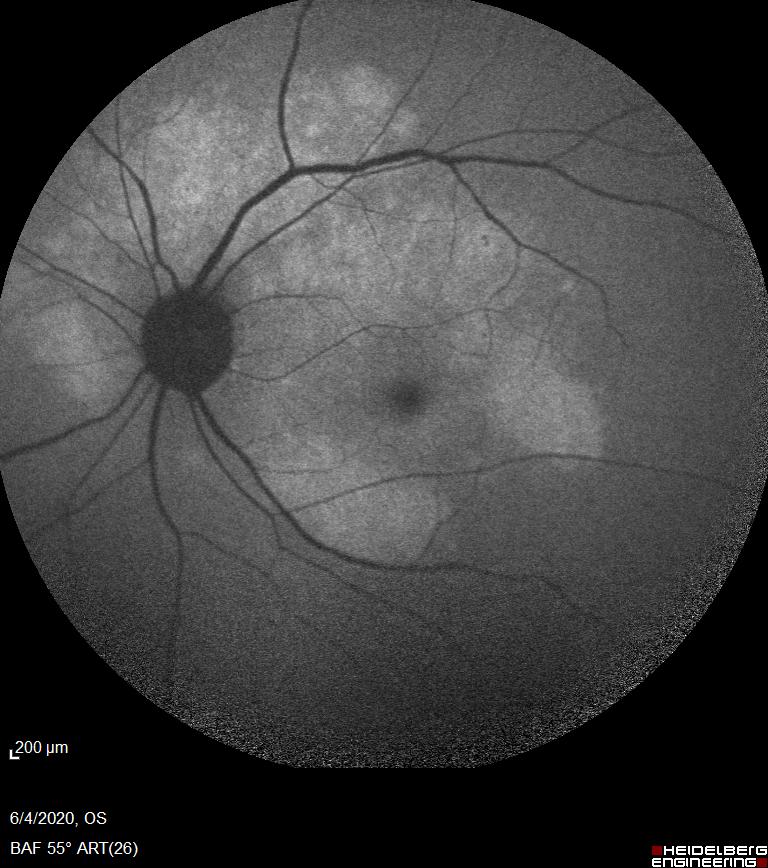
**

**Figure S2.** Fundus autofluorescence photo demonstrating area of the posterior placoid chorioretinopathy.
